# Encapsulation of Imidazole into Ce-Modified Mesoporous KIT-6 for High Anhydrous Proton Conductivity

**DOI:** 10.3390/molecules29133239

**Published:** 2024-07-08

**Authors:** Agata Tabero, Aldona Jankowska, Adam Ostrowski, Ewa Janiszewska, Jolanta Kowalska-Kuś, Agnieszka Held, Stanisław Kowalak

**Affiliations:** 1 Faculty of Chemistry, Adam Mickiewicz University, Uniwersytetu Poznańskiego 8, 61-614 Poznań, Poland; agata.tabero@amu.edu.pl (A.T.); aljan@amu.edu.pl (A.J.); eszym@amu.edu.pl (E.J.); jolakow@amu.edu.pl (J.K.-K.); awaclaw@amu.edu.pl (A.H.); 2 Institute of Molecular Physics, Polish Academy of Sciences, Smoluchowskiego 17, 60-179 Poznań, Poland

**Keywords:** proton conductivity, imidazole, cerium modification, Brønsted acid centers, KIT-6 porous materials

## Abstract

Imidazole molecules entrapped in porous materials can exhibit high and stable proton conductivity suitable for elevated temperature (>373 K) fuel cell applications. In this study, new anhydrous proton conductors based on imidazole and mesoporous KIT-6 were prepared. To explore the impact of the acidic nature of the porous matrix on proton conduction, a series of KIT-6 materials with varying Si/Al ratios and pure silica materials were synthesized. These materials were additionally modified with cerium atoms to enhance their Brønsted acidity. TPD-NH_3_ and esterification model reaction confirmed that incorporating aluminum into the silica framework and subsequent modification with cerium atoms generated additional acidic sites. UV-Vis and XPS identified the presence of Ce^3+^ and Ce^4+^ in the KIT-6 materials, indicating that high-temperature treatment after cerium introduction may lead to partial cerium incorporation into the framework. EIS studies demonstrated that dispersing imidazole within the KIT-6 matrices resulted in composites showing high proton conductivity over a wide temperature range (300–393 K). The presence of weak acidic centers, particularly Brønsted sites, was found to be beneficial for achieving high conductivity. Cerium-modified composites exhibited conductivity surpassing that of molten imidazole, with the highest conductivity (1.13 × 10^−3^ S/cm at 393 K) recorded under anhydrous conditions for Ce-KIT-6. Furthermore, all tested composites maintained high stability over multiple heating and cooling cycles.

## 1. Introduction

Proton exchange membrane fuel cells (PEMFCs) represent a significant advancement in hydrogen technology. These fuel cells are characterized by high energy efficiency, easy scalability, long operating times, and low (or zero) emissions. Fuel cell technology has applications in vehicles, stationary power generation, and portable devices [1].

One key material in PEMFCs is Nafion, a sulfonated fluoropolymer that serves as a state-of-the-art solid electrolyte. Nafion demonstrates an excellent proton conductivity of 10^−1^–10^–2^ S/cm at temperatures between 333 and 353 K in high humidity conditions [2,3]. However, its high production costs and complex synthesis processes have hindered its large-scale application [4]. Furthermore, Nafion’s conductivity dramatically decreases when temperatures exceed 353 K or drop below 268 K [3]. This decline is due to the collapse of the proton transport pathway at high temperatures and weak proton transfer in freezing environments [5]. Most solid electrolytes, including Nafion, suffer from a narrow operating temperature range and a heavy dependence on water for proton conduction, a characteristic explained by the Grotthuss mechanism [4]. Considering the necessity for PEMFCs to perform in diverse and sometimes extreme conditions, there is an urgent need for the development of stable electrolytes that can deliver high proton conductivity without reliance on water over a broad temperature range from subzero levels to above 373 K [3].

Water molecules are not the only molecules that can serve as proton carriers, as is the case with Nafion. Another possibility, for example, is to replace water with azoles, which can increase the maximum operating temperature of the fuel cell [6]. Among the studied alternative solid electrolytes, a very interesting candidate seems to be an inorganic–organic composite based on introduced imidazole (Im). Imidazole is a polar and amphoteric compound with low volatility (boiling point at ca. 523 K). In its solid crystalline form, imidazole exhibits low proton conductivity, which significantly increases upon its melting due to enhanced molecular mobility. Importantly, this increased mobility can also be achieved by dispersing imidazole within porous matrices, where its conductivity is substantially boosted [7]. So far, numerous materials, including MOFs [8], mesoporous solids [9], zeolites [10], or polymers [11], have been reported as matrices of imidazole-containing composites, and the number of publications on this subject continues to grow. It is worth noting, however, that among the studied proton conductors, materials containing water molecules still dominate, and the published results may inspire further research on anhydrous materials.

Studies conducted to date suggest that the conductivity of electrolytes, both hydrated and anhydrous, can be influenced by both chemical and structural properties of the porous material used. Among others, electrolytes based on porous silicates (including mesoporous silicas) [12,13] and aluminosilicates (e.g., zeolites) [14,15] have been studied. Materials with different pore arrangements (2D, 3D) [16,17,18] and different pore sizes (micropores, mesopores) [19] have been used. The introduction of a significant amount of proton carriers and the maintenance of their mobility may be facilitated by the large specific surface area and pore volume of the porous matrix. In this context, therefore, ordered mesoporous silicas seem attractive [20,21].

On the other hand, other studies have shown that the conductivity of electrolytes, e.g., those with imidazole, can be influenced by the parameters of the porous matrix, such as the number, type, and location of different types of active centers [22,23,24]. Mesoporous silicas usually have a significantly lower content of such centers than zeolites [25], but additional centers can be generated by the appropriate modification of the chemical composition of the silica either during its synthesis or as part of post-synthesis modification [20]. One of the most commonly used methods of increasing the acidity of mesoporous silicas is modification with sulfonic acid groups. The introduction of -SO_3_H generates additional protons, which can significantly increase the proton conductivity of electrolytes [13].

Another method of generating acidity is the modification of a mesoporous silica matrix with heteroatoms, including aluminum or cerium atoms. Depending on the method used to introduce cerium atoms, porous materials can vary in the type and number of active centers, among other factors [26,27]. Cerium atoms can be incorporated into both microporous materials (such as zeolite Y (FAU) [28,29], MOR [27], SAPO [26], BEA [30], MFI [30], FER [30], MCM-22 [31]) and mesoporous materials (e.g., MCM-41 [32,33], SBA-15 [34], MCM-48 [35], KIT-6 [36,37]). For microporous materials, ion exchange [28,31,38] and impregnation [26,39] are frequently the preferred modification methods. In contrast, for mesoporous materials, cerium is frequently introduced during one-pot synthesis [40,41] and via impregnation [32,37]. During ion exchange, cerium atoms can often be introduced as cations located in extra-framework positions [38], while incorporating cerium precursors into the reaction gel during mesoporous material synthesis can lead to their incorporation into the silica framework [34,41]. Conversely, when cerium atoms are introduced via impregnation, their further calcination in air can result in its deposition as an oxide phase (e.g., CeO_2_) on a silica surface [37,42]. It is also well known that during the thermal treatment of ion-exchanged cerium zeolites, additional Brønsted acid centers may be formed due to the decomposition of hydrated cerium species that increase the activity of the zeolites [38]. Therefore, the type of modification method chosen makes it possible to obtain materials with the desired properties [37,42]. Materials modified with cerium atoms have been used as catalysts [32,36] and adsorbents [33,43], among other applications.

In our research, KIT-6 mesoporous materials, characterized by a 3D structure with interconnected channels, a very large specific surface area (approximately 800 m^2^/g), and significant pore volume (up to 1.05 cm^3^/g) [44], were chosen as components for inorganic–organic composites with imidazole. Catalytic results from KIT-6 modified with cerium atoms incorporated into the mesoporous network confirm the presence of acidic centers in this system [36]. Therefore, this highly porous 3D system, with a potentially significant number of acidic sites, appears to be a promising candidate for proton conductor applications.

This study focuses on anhydrous proton-conducting materials derived from KIT-6 mesoporous materials and azoles, in which imidazole molecules play the role of the main proton carriers. The aim of the research was to investigate the effect of the acidity of KIT-6 materials on the conductivity of the obtained azole composites. The acid centers in KIT-6 were generated using two modification methods: (1) the introduction of aluminum into the KIT-6 framework at the synthesis stage of this material via the isomorphous substitution of silicon atoms with aluminum atoms; and (2) the introduction of cerium species as part of the post-synthesis modification of KIT-6 (Scheme S1). Both methods were expected to lead to an increase in the number of Brønsted acid centers and consequently an increase in the H^+^ content in the tested proton conductors. In the case of Al introduction into the framework, protons from the Brønsted acid centers would compensate for the negative lattice charge of KIT-6 generated by the insertion of aluminum atoms into the normally pure silica KIT-6, and the number of these centers could depend on the amount of aluminum in the material. This modification allowed us to achieve porous matrices with varying Si/Al ratios. Additionally, pure silica KIT-6 was also synthesized. Some of the resulting hydrogen forms of KIT-6 were then modified with cerium ions to generate additional acidic centers. It was assumed that Brønsted acid centers in Ce-KIT-6 materials could be formed as a result of the transformation of cerium species introduced into H-KIT-6 by ion exchange.

Both the cerium-modified and -unmodified (hydrogen) forms of KIT-6 materials were subjected to comprehensive physicochemical characterization, including X-ray diffraction (PXRD), transmission electron microscopy (TEM), Fourier-transform infrared spectroscopy (FTIR), UV-visible spectroscopy (UV-Vis), X-ray photoelectron spectroscopy (XPS), and low-temperature nitrogen adsorption. Furthermore, the proton conductivity of the materials with incorporated imidazole was evaluated using impedance spectroscopy (EIS). KIT-6 materials were found to be highly effective porous matrices for imidazole incorporation. The conductivity of the resulting composites ranged from 10^−4^ to 10^−3^ S/cm, placing them among the high-performing anhydrous electrolytes containing imidazole.

The studies presented here are an attempt to find a correlation between the acidity of the porous host and the conductivity of anhydrous proton conductors. Although the idea of introducing cerium atoms, e.g., into heterogeneous catalysts, has been known for many years, the attempt to create Bronsted acid centers by introducing cerium species to generate additional H^+^ in the proton conductor can definitely be considered as an unconventional approach to such studies. In the case of the presented studies, it should also be emphasized that the introduction of imidazole into silica-based mesoporous materials is still not very common in the literature. For example, the use of KIT-6 materials in proton conductors has so far been mainly limited to the use of materials with sulphonic acid groups, and in all of them, to the best of our knowledge, water molecules have played the role of the main proton carriers [16]. Therefore, studying anhydrous systems containing Ce-modified KIT-6 in terms of proton conductivity can provide valuable information about these relatively unexplored materials.

## 2. Results

### 2.1. Structure

**Figure 1:**
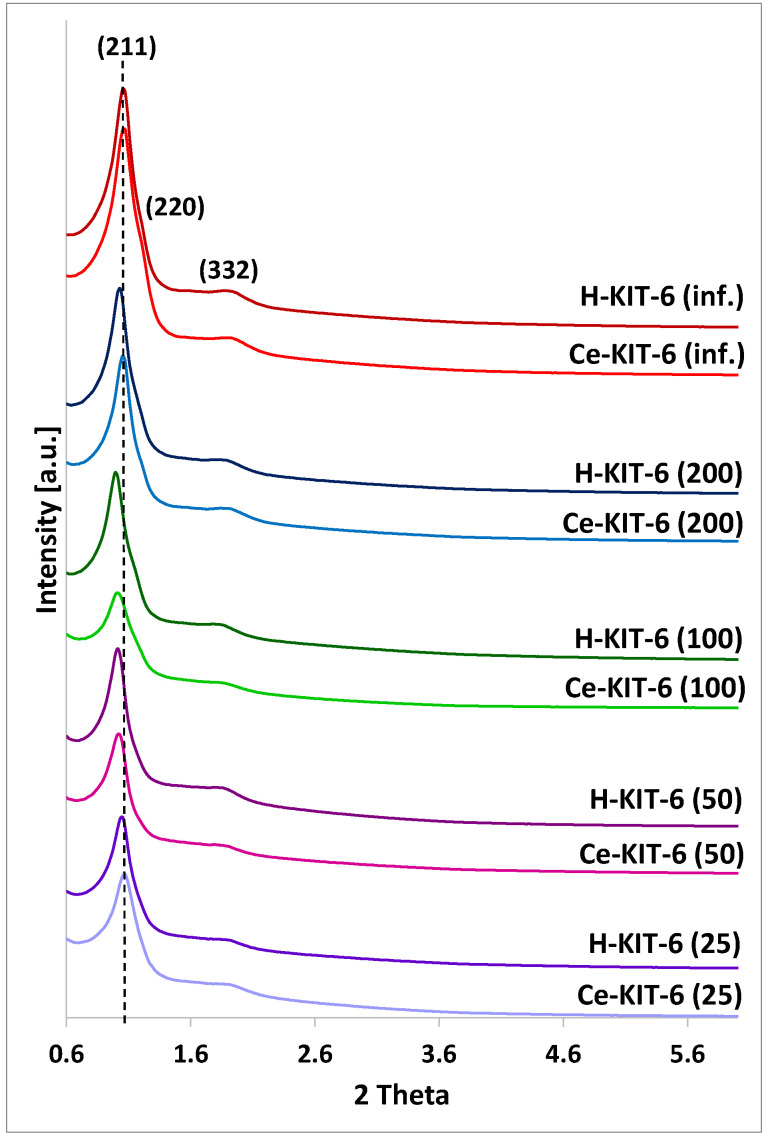
PXRD patterns of KIT-6 matrices with different Si/Al ratio: 25; 50; 100; 200; and infinity.

All the obtained materials exhibit reflections (211), (220), and (332), characteristic of the KIT-6 structure with a cubic space group Ia3d symmetry (Figure 1) [45,46,47]. As the amount of aluminum introduced during synthesis increases, the intensity of the reflections decreases, indicating a structural modification of the materials and some reduction in the ordering of their structure. A similar decrease in reflection intensity is observed after the introduction of cerium, suggesting a disruption of the ordered structure of the modified materials. This could be attributed to the use of cerium chloride salt as the cerium source, where the remaining chloride ions after modification interact with the silica framework during thermal treatment, resulting in the formation of structural defects that can serve as acid sites. Similar observations have been reported for amorphous disordered silica [48] or crystalline zeolite-structured silicas [49].

Additionally, the introduction of both aluminum and cerium results in a visible shift of the (211) reflection towards lower 2Theta values. This shift is associated with an increase in the interplanar spacing d(211). The unit cell parameter for all matrices was calculated using the formula a_0_ = √6 × d_211_ (Table 1). The wall thickness (d_s_), derived using d = (a/2) − D (where D is the pore diameter and a is the unit cell parameter), is also presented in Table 1 for comparison.

The presence of both aluminum and cerium affect the values of the unit cell parameter and the wall thickness of the obtained materials. With an increasing amount of aluminum, we initially observed an increase in the unit cell parameter and wall thickness (up to Si/Al = 100), followed by a decrease for samples with Si/Al = 50 and 25. The observed increase in interplanar spacing with rising aluminum content may indicate the incorporation of aluminum into the framework. Since the Al-O bond length is 1.761 Å and the Si-O bond length is 1.603 Å, aluminosilicate materials show higher unit cell parameter values compared to purely siliceous materials [50,51,52]. The decrease in the unit cell parameter and wall thickness for materials with the highest aluminum content may be related to a decrease in the ordering of these materials. A similar tendency between the a_0_ or d-spacing and heteroatoms content was also presented in the literature [36].

For all cerium forms, in comparison to their hydrogen counterparts, we observe both a reduction in wall thickness and a decrease in the unit cell parameter a_0_, which may result from a decrease in the ordering of these materials.

### 2.2. Acidic Properties of the Studied Materials

TPD-NH_3_ analysis was performed to determine the quantity and strength of acid sites in KIT-6 generated mainly by using two modification methods: (1) the introduction of aluminum into the KIT-6 framework; and (2) the introduction of cerium species as part of the post-synthesis modification of KIT-6. The desorption maxima for H-KIT-6 and Ce-KIT-6 materials were recorded below 653 K, which suggests that these materials possess only weak acid sites (Table 2). The number of acid sites in the obtained samples is relatively small and increases with increasing aluminum content. The subsequent modification of Al-containing KIT-6 with cerium results in a significant increase in the number of acid sites. However, no linear correlation was observed between the Si/Al ratio and the number of acid sites for Ce-containing samples. Initially, the number of acid sites increases with increasing aluminum content up to Si/Al = 100 and then decreases for samples with higher Al loading (Si/Al = 50 and 25). The highest number of acid sites is found in the Ce-KIT-6 (100) sample. Additionally, there is a noticeable broadening of the peaks and a shift in the ammonia desorption maxima towards higher temperatures, indicating a slight increase in the strength of the acid sites (Figure S1).

The density of acid sites, expressed as the number of acid sites [μmol] per m² of the specific surface area of the KIT-6 materials, clearly indicates that cerium modification generates additional active sites (Table 3), which is in accordance with the TPD-NH_3_ measurements. For Ce-containing samples, the concentration of acid sites increases with an increase in the Si/Al ratio, achieving its maximum for the Ce-KIT-6 material with a Si/Al ratio of 100. A somewhat different trend was observed for the H forms of KIT-6, where the number of acid sites corresponds to the amount of aluminum ions introduced during the synthesis of these materials. Hence, the highest density of acid sites is observed in the H-KIT-6 material with the lowest Si/Al ratio of 25.

The presence of acid sites was further confirmed by activity in the esterification reaction of acetic acid (HAc) with methanol (Table 4). Both hydrogen- and cerium-containing materials exhibited activity in the acid-catalyzed reaction. For H-forms, HAc conversion increased with increasing Si/Al ratio, confirming the presence of Brønsted acidic centers generated by the aluminum atoms located in the framework of KIT-6 and compensated by protons. The introduction of cerium into KIT-6 materials further enhanced their catalytic activity in the esterification reaction. Among the cerium-containing forms, the highest degree of HAc conversion was observed for the Ce-KIT-6 (100) sample, while the lowest activity showed Ce-KIT-6 (inf.). These results strongly correlate with the number and density of acidic sites (Table 2 and Table 3).

### 2.3. Textural Properties of KIT-6 Materials

**Figure 2:**
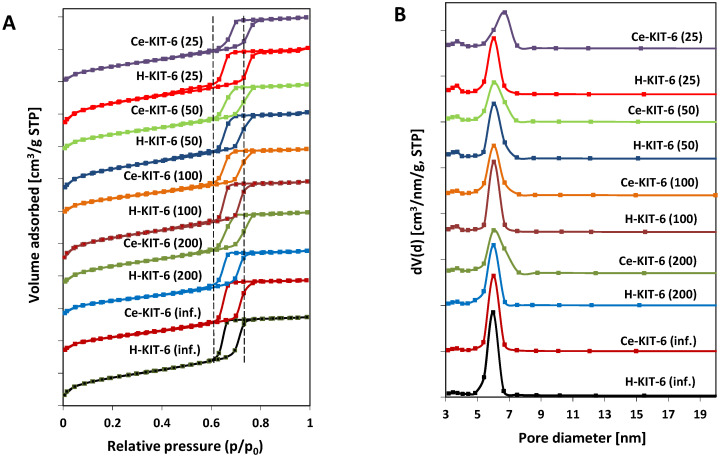
N_2_ adsorption/desorption isotherms (**A**) and pore size distribution (**B**) of KIT-6 materials.

The N_2_ adsorption–desorption isotherms of both hydrogen- and cerium-containing KIT-6 materials exhibit a type IV characteristic for mesoporous materials, with capillary condensation occurring between 0.6 and 0.8 *p/p*_0_ and a distinct final saturation plateau (Figure 2A). The presence of an H1 hysteresis loop indicates that the obtained materials have large pores within a narrow size range (Figure 2B). The BET surface area, pore volume, and pore diameter values of the obtained materials are presented in Table 5. All samples exhibit pore diameters ranging from 4.1 to 5.2 nm and large BET surface areas ranging from 627 to 922 m^2^/g. With increasing aluminum content, we observe an increase in specific surface area and a decrease in average pore diameter. Modification with cerium cations, however, leads to a reduction in the surface area of the materials by over 250 m^2^/g (for Ce-KIT-6 (100) and Ce-KIT-6 (50)), 237 m^2^/g for Ce-KIT-6 (25), and approximately 150 m^2^/g for high silica materials. In the case of Ce-modified KIT-6, the contribution of micropore surface area (S_micro_) also decreases with increasing aluminum content. For the sample with Si/Al = 25, the S_micro_ decreases by nearly 150 m^2^/g, while for the pure silica sample (Si/Al = infinity), it decreases only by 2 m^2^/g. Cerium modification also reduces the pore volume by approximately 0.1–0.2 [cm^3^/g], confirming the introduction of cerium species into the pores, and increases the average pore diameter by 0.4–0.6 nm. Smaller changes in textural properties observed in cerium-containing high-silica materials (KIT-6 (inf.) and KIT-6 (200)) may indicate a lower amount of cerium introduced into their pores.

As shown, most samples exhibit nearly vertical adsorption branches in the capillary condensation region, characteristic of materials with well-defined internal pore sizes (Figure 2A). However, for Ce-containing forms, there is a slight shift and broadening of the hysteresis loop profile, indicating wider pore sizes and a reduced uniformity of the pore system, respectively. These observations are consistent with pore size distribution analysis (Figure 2B). Comparing the pore size distribution profiles of the pure-silica or aluminosilicate materials with their cerium-modified counterparts reveals a decrease in the contribution of the smallest pores and an increase in larger pores. This suggests that micropores are partially blocked by cerium species and that the KIT-6 structure is modified with chloride ions, thereby increasing porosity by removing some heteroatoms from the framework.

### 2.4. Infrared Spectroscopy

**Figure 3:**
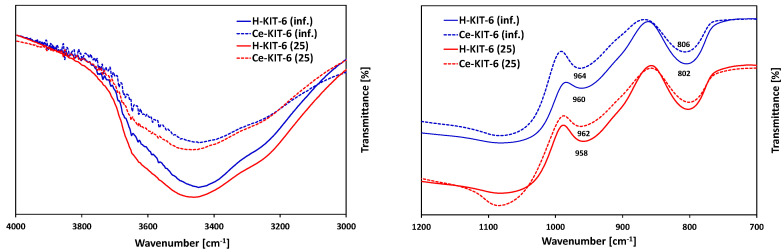
FTIR spectra of selected H-KIT-6 and Ce-KIT-6 samples with different Si/Al ratio.

FTIR analysis in the range of 4000–400 cm^−1^ (Figure 3) revealed absorption bands present in all samples at ~3450, 1630, 1200, 1075, 960, 800, and 460 cm^−1^. The band at 3450 cm^−1^ is attributed to stretching vibrations of the surface and bridging hydroxyl groups ν(Si-O-H) [53,54], whereas the band at 1630 cm^−1^ is associated with physically adsorbed water [54]. Bands in the range of 1000–1250 cm^−1^ originate from stretching vibrations ν_as_(Si-O-Si), while the band at 800 cm^−1^ corresponds to ν_s_(Si-O-Si) vibrations [55]. The band at approximately 960 cm^−1^ is related to stretching vibrations of silanol groups ν_as_(Si-OH) [56], while the band at 460 cm^−1^ is assigned to bending vibrations of δ(Si-O-Al) bonds [57].

FTIR analysis is commonly used to confirm the presence of introduced heteroatoms into or outside the framework of silica-based porous materials. When cerium is incorporated into the framework, it forms only siloxane bridges Si-O-Ce^4+^ without attached OH groups. However, if cerium is localized in an extra-framework position, Ce-OH groups are formed, whose vibrations are observed in the range of 3660–3675 cm^−1^ [39]. FTIR spectra recorded for H-KIT-6 and Ce-KIT-6 samples in the 3000–4000 cm^−1^ range are depicted in Figure 3A. Due to the low concentration of cerium atoms and their overlapping with the Si-OH group, confirming the presence of the bands attributed to Ce-OH vibrations is challenging. However, it is noticeable that the intensity of this band decreases upon cerium introduction. According to Timofeeva, this phenomenon may result from cerium introduced into the KIT-6 framework [34]. The presence of large cerium atoms could cause the removal of OH groups and the formation of more defective sites [34].

Since cerium chloride was used as the cerium precursor, its presence could potentially lead to the removal of framework atoms and the creation of defects, where cerium is subsequently incorporated during thermal treatment. This formation of defects is well-documented in the literature and often utilized for isomorphic substitution [48,49,58]. According to the literature, the introduction of cerium into the MCM-41 framework typically results in shifts of bands around 950–980 cm^−1^ and 800–820 cm^−1^ [59,60,61]. Similarly, in the presented spectra, we observe small shifts (approximately 5 cm^−1^); however, these shifts are not pronounced enough to provide definitive evidence of cerium incorporation into the structure. Furthermore, according to the literature, the band associated with Si-O-Ce vibrations at 970 cm^−1^ overlaps with the bands from Si-O-Si vibrations at 966 cm^−1^ and from Si-OH group vibrations at 960 cm^−1^ [59,60].

### 2.5. UV-Vis Analysis

**Figure 4:**
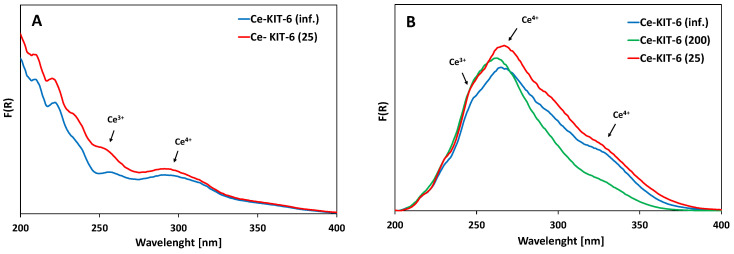
UV-Vis spectra of selected Ce-KIT-6 materials before (**A**) and after calcination at 673 K (**B**).

The UV-Vis spectra of all Ce-KIT-6 materials exhibit broad and intense absorption bands in the range of 250–350 nm, commonly attributed to charge transfer transitions involving O-Ce (Figure 4). The analysis of these spectra is challenging due to the presence of wide bands and reflection effects commonly observed in the spectra of cerium-containing materials [62,63]. Charge transfer transitions of Ce^3+^ ions are typically observed in the UV region at ca. 200–250 nm [64], whereas transitions of Ce^4+^ ions occur around 280 nm [65]. The band at approximately 340 nm is assigned to a charge transfer transition of Ce 4f-O 2p between cerium 4f orbitals and oxygen 2p orbitals [66,67]. Bulk CeO_2_ bands are visible above 400 nm [34,59].

In all samples modified with cerium, bands corresponding to both Ce^3+^ and Ce^4+^ were identified (Figure 4). UV-Vis analysis did not show any correlation between the Si/Al ratio and the intensity of these cerium-related bands. It is clear that the spectra of the materials changed significantly after calcination following cerium introduction. When cerium is introduced into the porous material as an exchangeable cation, a band around 250 nm, accompanied by a less intense band at approximately 300 nm, is observed. Upon high-temperature treatment, additional intense bands appear at 280 nm and 340 nm, which align with findings in the literature and are attributed to cerium cation dehydration [31,68]. According to Gil, this could also be due to the formation of small cerium oxide clusters [68]. However, no additional bands indicating extra-framework CeO_2_ forms were observed in the 350–800 nm range for any of the samples. Moreover, PXRD analysis conducted in the wide 6–60° 2Theta range (Figure S2) did not reveal any additional oxide phases; instead, only a broad band characteristic of amorphous silica was observed. [34,69]. However, it cannot be ruled out that the amount of cerium oxide is too low or is highly dispersed, which may hinder its detection by PXRD analysis.

### 2.6. TEM Analysis

**Figure 5:**
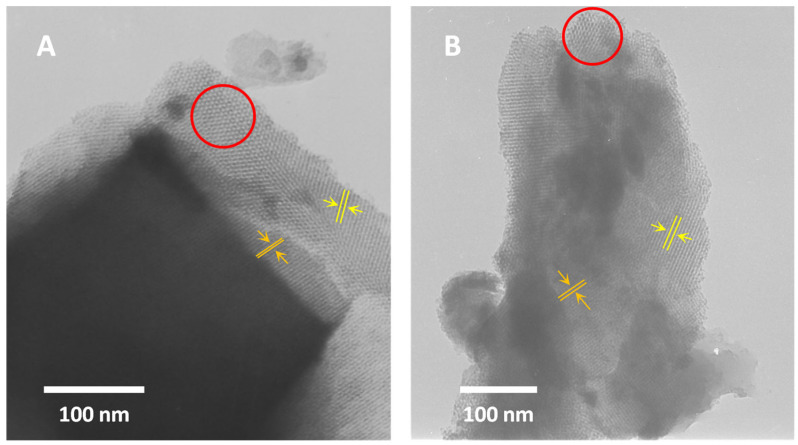
TEM images of H-KIT-6 (100) (**A**) and Ce-KIT-6 (100) (**B**) samples.

To assess the texture of KIT-6 materials, transmission electron microscopy (TEM) was employed. TEM analysis confirmed the absence of CeO_2_. Additionally, it is evident that cerium modification does not alter the ordering of the samples. Images of both materials after synthesis (Figure 5A) and after cerium treatment (Figure 5B) exhibit similar features typical of a three-dimensional cubic structure [44,70,71,72]. They clearly show the characteristic honeycomb structure (highlighted in red circle) and parallel lines with varying spacings.

### 2.7. XPS

**Figure 6:**
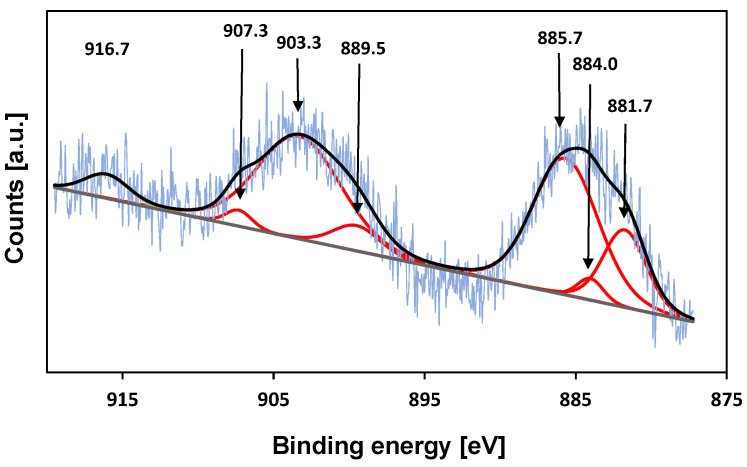
XPS spectrum of the Ce3d core level of Ce-KIT-6 (100).

To further investigate the oxidation state of cerium on the surface of cerium-treated KIT-6 samples, X-ray photoelectron (XPS) measurements were carried out (Figure 6) The binding energies in the range of 881–886 eV shown in the spectra Ce-KIT-6 (100) corresponding to Ce3d_5/2_ and its satellite and the peaks in the range of 900–907 eV corresponding to Ce3d_3/2_ and its satellite indicate that some part of Ce ions remain trivalent after calcination at a temperature of 673 K. However, the presence of a peak at a binding energy of ~917 eV, characteristic of Ce^4+^ species, suggests that some of the cerium ions have been oxidized to tetravalent species during thermal treatment [39,71].

### 2.8. Proton Conductivity

Conductivity measurements were performed on composites based on KIT-6 type matrices containing various concentrations of imidazole (ranging from 0.29 to 0.6 mass fraction). These measurements involved two heating–cooling cycles between 300 and 393 K, with a temperature ramp of 1 K/min. The same experimental setup was used for polycrystalline imidazole and matrices H-KIT-6 and Ce-KIT-6 without imidazole filling.

**Figure 7:**
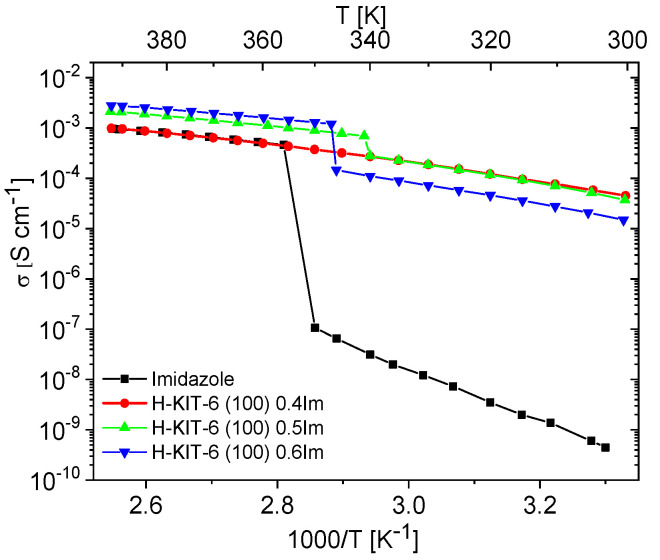
Temperature dependence of conductivity measured during the second cooling cycle for imidazole and imidazole-containing H-KIT-6 composites of various Im loading.

**Figure 8:**
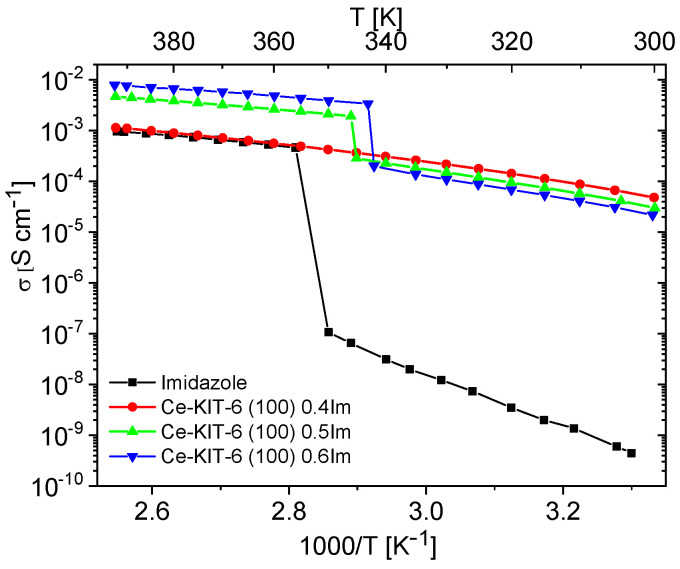
Temperature dependence of conductivity measured during the second cooling cycle for imidazole and imidazole-containing Ce-KIT-6 composites of various Im loading.

Unfilled matrices exhibited low and notably unstable conductivity values, which decreased significantly after the first heating cycle due to the removal of water molecules from the materials. For all the tested matrices, conductivity at 393 K during the second heating cycle reached maximum values around 10^−9^ S/cm. This was approximately six orders of magnitude lower compared to the investigated composites and pure imidazole. Imidazole, a solid at room temperature, also displayed very low conductivity (around 10^−8^–10^−9^ S/cm), attributed to the restricted mobility of azole molecules within the solid-phase crystalline network. Previous studies by Kawada have interpreted conductivity measurements for polycrystalline imidazole [73]. As temperature increases, its conductivity gradually rises and then sharply increases by several orders of magnitude upon melting. In the liquid phase, there is a significant enhancement in molecular mobility and reorientation capability of imidazole molecules. This phase also facilitates the formation and breaking of hydrogen bonds between adjacent molecules, resulting in effective proton transport and a notable increase in proton conductivity [10,19,21,23]. A similar liquid imidazole conductivity enhancement can be achieved by dispersing imidazole molecules within the pores of molecular sieves. Our results demonstrate that composites prepared in this manner can exhibit conductivity up to five orders of magnitude higher than solid-phase imidazole at room temperature (Figure 7 and Figure 8).

The conductivity measurements for composites containing varying concentrations of imidazole are shown in Figure S3. These measurements aimed to identify the maximum concentration at which imidazole remains dispersed within the matrix. Initially, as the concentration of imidazole increased up to 0.4 mass fraction, the conductivity of the composites steadily rose. However, for composites with concentrations of 0.5 and 0.6 mass fractions, a phase transition effect (melting/freezing) similar to pure imidazole was observed (refer to Figure 7 and Figure 8, Figure S3). This suggests that at such a high concentration of imidazole, further dispersion within the pores is not feasible. Instead, imidazole molecules form aggregates, resembling the polycrystalline imidazole phase on the matrix surface, as shown by PXRD studies (Figure S4).

To understand the high conductivity of the Ce-KIT-6 (100) composite containing 0.5 and 0.6 Im, it is crucial to consider the properties of bulk imidazole in the liquid phase. Imidazole molecules in this phase can translate and perform a range of movements, including rotations and oscillations. As a result, the vehicular mechanism or the Grotthuss conduction mechanism can occur. In the efficient two-step Grotthuss mechanism, an effective proton hoping within the hydrogen bond of the Im-Im dimer is required, followed by its transfer between neighboring molecules. Consequently, besides fast molecular dynamics, imidazole molecules should exhibit high structural order, which increases the likelihood of efficient proton transfer between neighboring molecules and, thereby, enables an efficient conduction pathway. In porous materials, imidazole molecules can be dispersed closely together in regular and structurally equivalent positions, which increases the probability of effective proton transfer compared to the liquid state, where the molecules are less organized and their movements are more chaotic. Spatial constraints within the pores can also reduce the random and chaotic translational movements of imidazole molecules that occur in the liquid phase, leading to more coordinated translation (charge diffusion), which is required in the vehicular mechanism.

**Figure 9:**
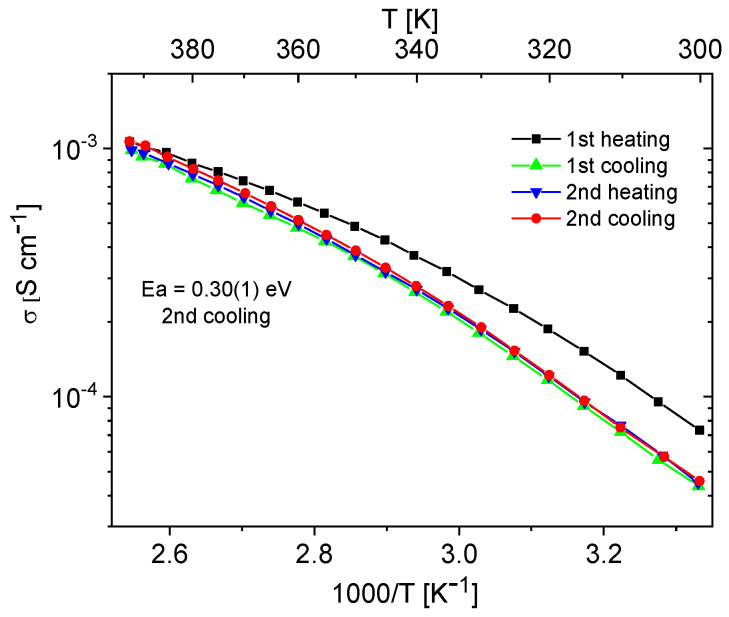
Temperature dependence of conductivity for the H-KIT-6 (100) 0.40 Im composite recorded for two heating–cooling cycles.

**Figure 10:**
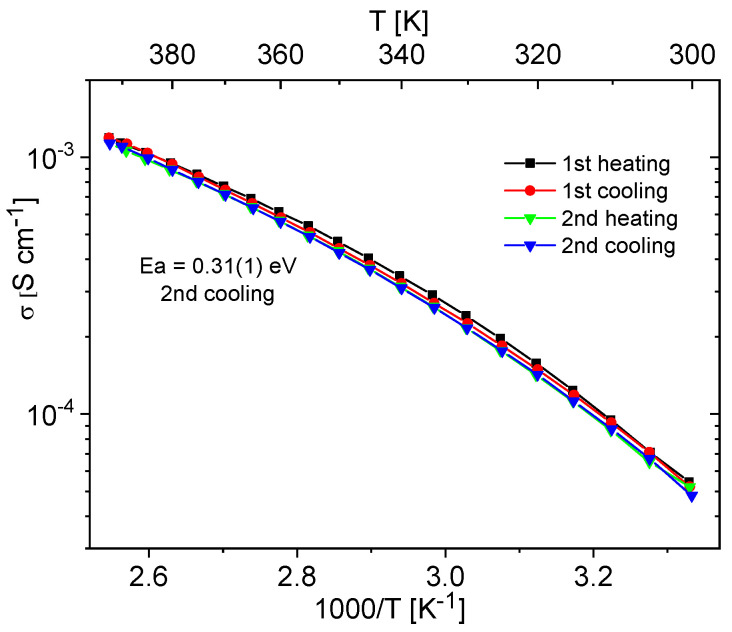
Temperature dependence of conductivity for the Ce-KIT-6 (100) 0.40 Im composite recorded for two heating–cooling cycles.

Materials containing 0.4 Im exhibited the best proton-conducting properties; therefore, they were extensively discussed in the article. All composites, both in the hydrogen- and cerium-modified forms, demonstrated high and stable conductivity within the investigated temperature range (300–393 K). Figure 9 and Figure 10 illustrate a typical temperature dependence of conductivity. The graphs indicate individual heating and cooling cycles, as well as the activation energy value obtained from the second cooling cycle. It is noticeable that there is a slight decrease in conductivity between the first heating cycle and the second heating cycle, but during the second heating–cooling cycle, the conductivity remains stable.

**Figure 11:**
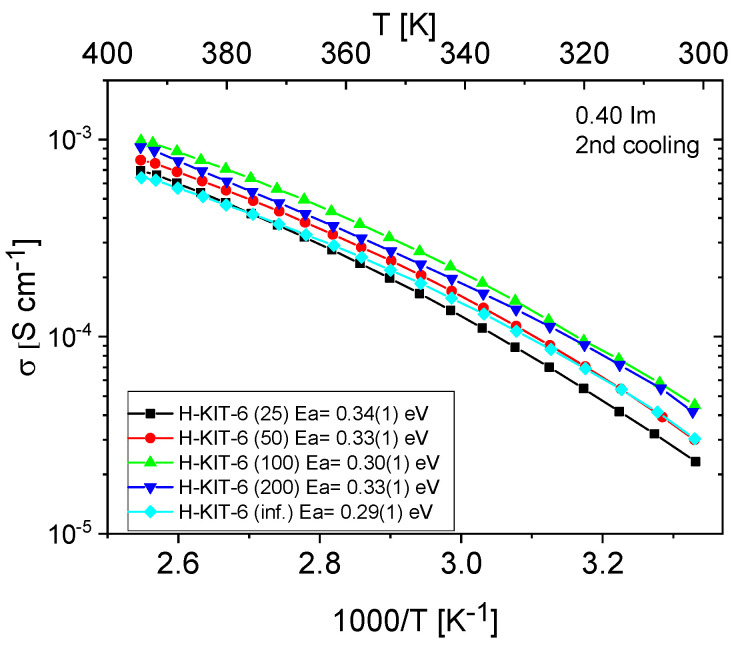
Temperature dependence of conductivity and activation energies for H-KIT-6 composites with different Si/Al ratios measured during the second cooling cycle.

**Figure 12:**
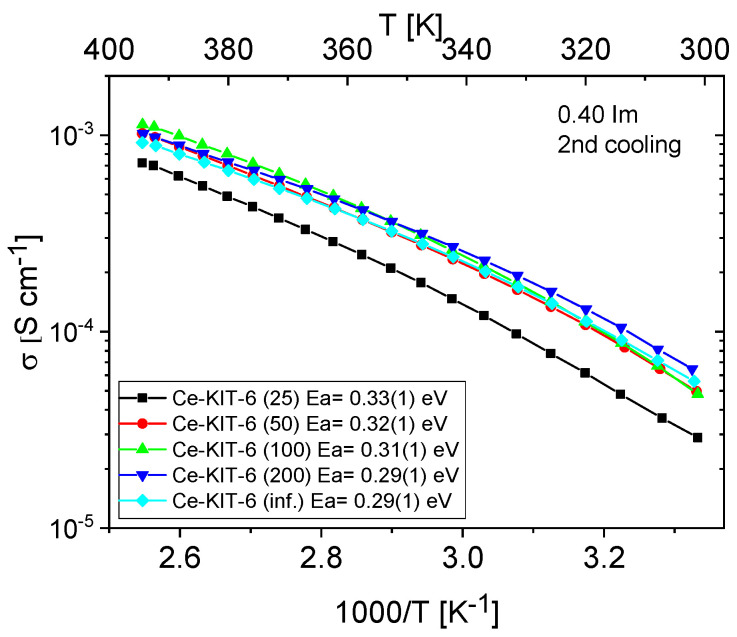
Temperature dependence of conductivity and activation energies for Ce-KIT-6 composites with different Si/Al ratios measured during the second cooling cycle.

Figure 11 and Figure 12 show the temperature-dependent conductivity of all composites with varying Si/Al ratios, measured during the second cooling cycle. Throughout this cycle, both H-KIT-6- and Ce-KIT-6-based forms of all composites exhibited consistently high and stable conductivity within the investigated temperature range (300–393 K). For all composites, conductivity increased with rising temperature and aluminum content. However, composites with the highest aluminum content (Si/Al = 50 and 25) showed a decrease in conductivity. Moreover, the cerium-modified KIT-6 forms demonstrated higher conductivity compared to H-KIT-6, indicating that introducing cerium into the initial matrix positively influences composite conductivity.

**Figure 13:**
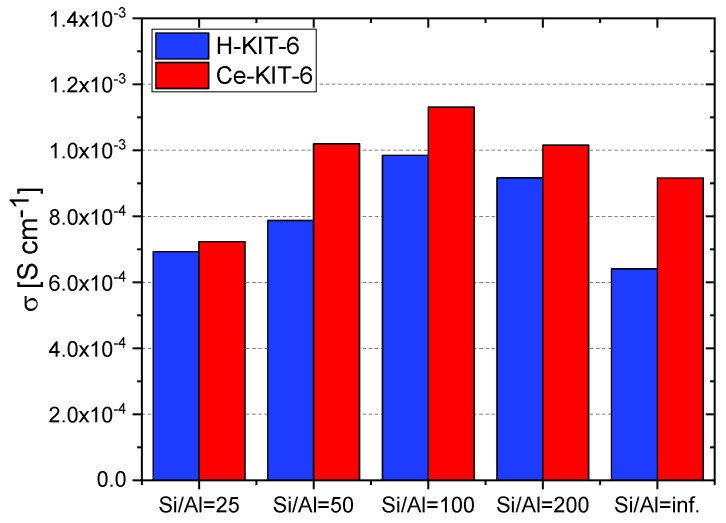
Conductivity values of selected composites determined at 393 K for second cooling cycle.

At 393 K, the highest conductivity (1.13 × 10^−3^ S/cm) was recorded for the Ce-KIT-6 (100) composite, and slightly lower values (1.02 × 10^−3^ S/cm) were obtained for Ce-KIT-6 (50) and Ce-KIT-6 (200) composites (Figure 13). Importantly, their conductivity was even higher than that of molten imidazole. This demonstrates that the dispersion of imidazole molecules in a molecular sieve with a KIT-6 structure is an effective method for obtaining composites with favorable proton-conducting properties.

For comparison in Table 6, the proton conductivity values of azole-loaded composites are presented. As can be seen, all the composites listed exhibit lower proton conductivity than fully hydrated Nafion (which achieves 0.113 S/cm for Nafion membrane at 298 K) [2,3]. However, Nafion requires continuous hydration for stable and efficient operation and operates only within a very limited temperature range. In contrast, our studies closely resemble the conductivity values obtained for other nanoporous matrix-based electrolytes, especially anhydrous ones. Furthermore, the proton conductivity of Ce-KIT-6 (100)-0.4 Im is comparable to that of some MOF–azole systems for which experimental fuel cells have already been constructed [3].

Activation energy values were also determined from the conductivity measurements. For all composites, these values are 0.3 eV, which is very close to that of molten imidazole (about 0.24 eV), indicating a favorable dispersion of imidazole and weak interactions of imidazole molecules with the matrix resulting from a small number of weak centers in KIT-6.

Our previous studies, mainly on microporous composites, have demonstrated that the nature and number of acidic sites can significantly influence the conductivity of the resulting composites. These studies showed that using polar matrices containing acidic sites is more advantageous [10,24]. Composites based on non-polar matrices often exhibited high conductivity during the first measurement cycle but significantly lower values in subsequent cycles. This decrease in conductivity was attributed to the loss of weakly bound azoles (which exhibit hydrophilic properties) from the hydrophobic surface of the non-polar matrix. Furthermore, the studies indicated that the presence of strong acidic sites could impair conductivity, as strong interactions between the matrix and imidazole molecules can hinder their mobility.

**Figure 14:**
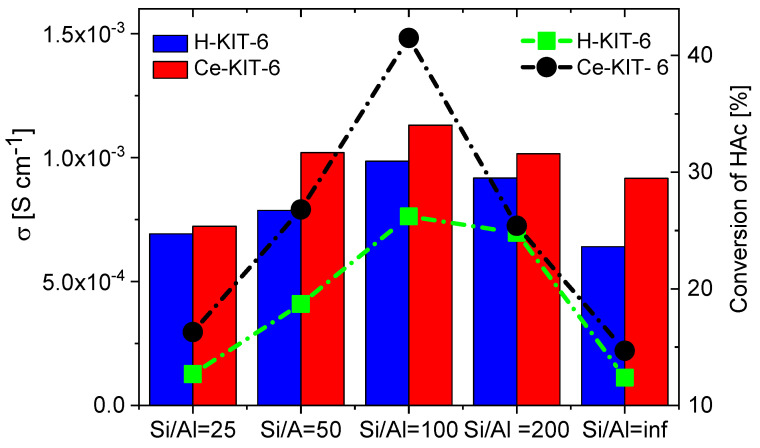
Correlation of conductivity values of selected composites determined at 393 K for second cooling cycle with acetic acid (HAc) conversion.

TPD-NH_3_ analysis revealed that all investigated matrices possess only weak acidic sites. For H-KIT-6-type matrices, the number of acidic sites and consequently their concentration is very low. However, this number increases for cerium-modified forms, which may explain the higher conductivity observed in these composites. Additionally, the presence of Brønsted acidic sites was confirmed through the esterification reaction of acetic acid with methanol (Table 3). Both the hydrogen and cerium forms exhibited activity in this reaction. For H-KIT-6 samples, activity increased with increasing Si/Al ratio, whereas matrices containing cerium showed even higher activity. Regardless of the cationic form, KIT-6 (100) exhibited the highest degree of conversion, while KIT-6 (25) and KIT-6 (inf.) showed the lowest. These results correlate with EIS measurements, where the highest conductivity was recorded for composites based on KIT-6 (100), and the lowest for KIT-6 (25) and KIT-6 (inf.) (Figure 14).

## 3. Conclusions

The synthesis of mesoporous molecular sieves with a KIT-6 structure and varying aluminum content demonstrated that the incorporation of aluminum into silica structures generates additional acidic sites, with their number increasing as the aluminum content rises. Additionally, the introduction of cerium cations significantly boosted the number of acidic sites. The catalytic activity of the matrices, confirmed by the esterification reaction of acetic acid with methanol, indicated the presence of Brønsted acidic sites, which are related to the presence of cerium atoms. UV-Vis and XPS analyses verified the presence of Ce^3+^ and Ce^4+^ in the KIT-6 materials, and the results suggest that high-temperature treatment after cerium introduction may lead to the partial incorporation of cerium into the framework.

EIS studies showed that dispersing imidazole in the KIT-6 matrices resulted in highly conductive composites, demonstrating that H^+^ transport in these composites is facilitated by imidazole molecules. All composites, both in their hydrogen and cerium forms, exhibited high conductivity within a wide temperature range (300–393 K). Measurements over several heating and cooling cycles confirmed that the samples maintained high and stable conductivity in subsequent cycles.

The cerium-modified KIT-6 materials provided higher conductivity in composites than their hydrogen counterparts, indicating that cerium incorporation positively impacts composite conductivity by increasing the number of protons. At 393 K, the highest conductivity under anhydrous conditions (1.13 × 10^−3^ S/cm) was recorded for the Ce-KIT-6 (100) composite, with slightly lower values (1.02 × 10^−3^ S/cm) observed for Ce-KIT-6 (50) and Ce-KIT-6 (200) composites. Remarkably, their conductivity was even higher than that of molten imidazole.

The presence of weak acidic sites, particularly Brønsted sites, positively affects proton conduction. Using matrices containing such sites leads to composites with high conductivity. Controlling the number, strength, and nature of these sites enables the creation of composites that can effectively compete with other nanoporous matrix-based electrolytes containing imidazole.

## 4. Materials and Methods

### 4.1. The Synthesis of KIT-6 Materials

Siliceous and aluminosilicate KIT-6 materials were synthesized by the assembly of polymer surfactant micelles as template under mild acidic conditions. A typical procedure for the synthesis was as follows: 4 g of Pluronic P123 (Sigma Aldrich, Poznań, Poland) was dissolved in 114 mL H_2_O and 39.2 mL 2 M HCl (Stanlab, Lublin, Poland). The mixture was stirred at 313 K until a clear solution was obtained (about 4 h). Then, 6.22 mL n-butanol (Eurochem BGD, Tarnów, Poland) was added and the stirring was continued for 1 h. After this time, 12.84 g of TEOS (Sigma Aldrich) and appropriate amounts of aluminum isopropoxide (Al(isop)_3_, Acros), given in Table 7, were added, and stirring was continued for 20 h at 313 K. Subsequently, the mixture was aged at 368 K for 24 h under static hydrothermal condition. The precipitated material was filtered, washed, and calcined in air at 823 K for 8 h to remove the template. The final materials were marked as H-KIT-6 (x) where x denotes Si/Al molar ratio of the gel.

### 4.2. The Preparation of Ce-KIT-6

For all H-KIT-6 materials, regardless of the Si/Al ratio, the same procedure for introducing cerium ions was used. A quantity of 5× *g* of the material was immersed in 50 mL of 0.1 M CeCl_3_ solution and left at room temperature for 2 h. After this time, the solution was centrifuged. The remaining sediment was immersed again in 50 mL of 0.1M CeCl_3_ solution and left for another 2 h. The sediment was centrifuged again and immersed in a new portion of the cerium chloride solution and then it was left for 20 h. After this time, the sediment was washed with 400 mL of H_2_O, dried, and then calcined for 2 h at 673 K. The procedure described above was performed three times. The resulting cerium form was labeled as Ce-KIT-6.

### 4.3. The Preparation of KIT-6/Imidazole Composites

Imidazole was introduced into 1 g of the matrix by the impregnation method. Before impregnation, the matrices were calcined at 623 K to remove water. Imidazole solutions in chloroform (3.4 mL) were added to the activated KIT-6 materials. The weights and mass fractions are listed in Table 8. The same weights were used for both the hydrogen and cerium forms. The mixture was stirred on a magnetic stirrer at room temperature for 24 h in closed vials. After this time, the solvent was evaporated at room temperature. The mass fraction of imidazole in the composite was labeled as x Im.

### 4.4. Characterization

PXRD measurements were performed using powder X-ray diffraction on a BRUKER D8 ADVANCE diffractometer (Bruker, Billerica, MA, USA) equipped with a Cu lamp (Cu Kα1) emitting radiation with a wavelength of λ = 0.15406 nm. The analysis was conducted in the small-angle (0.6–8°) and the wide-angle (6–60°) ranges.

N_2_ adsorption/desorption studies were conducted using a Quantachrome NOVA 1000 instrument (Anton Paar, Warsaw, Poland). Prior to measurement, the samples were degassed at a temperature of 393 K for 16 h. The surface area was calculated using the Brunauer–Emmet–Teller (BET) method. The total pore volume and the average pore diameter were determined by the Barrett–Joyner–Halenda (BJH) method.

The FTIR spectra were recorded using a Bruker Tensor 27 spectrophotometer (Bruker Polska, Poznan, Poland). Measurements were performed using the transmission technique in the wavenumber range of 4000–400 cm^−1^ with a resolution of 1 cm^−1^. The sample was mixed with 200 mg of KBr and formed into pellets using a press (150 MPa).

The temperature-programmed desorption of ammonia (TPD-NH_3_) was conducted using a PulseChemiSorb 2705 apparatus (Micromeritics, Norcross, GA, USA) with a flow system. The samples were pretreated in helium at a temperature of 823 K for 0.5 h. Gaseous ammonia was adsorbed at 393 K. The physically adsorbed NH_3_ was removed by purging with helium flow at 393 K for 1 h. Then, thermal desorption was carried out up to 923 K with a heating rate of 10 K/min. The amount of desorbed ammonia was measured using a TCD detector and recalculated per 1 g of sample.

The esterification reaction of acetic acid with methanol was performed in sealed glass vials. For this reaction, 0.05 g of catalyst, which had been pre-activated in an oven at 623 K for 1 h to remove water from the catalyst’s pores, was used. To the weighed catalyst, 1.501 g of glacial acetic acid (Carlo Erb, Cornaredo, Italy) and 1.602 g of methanol (Eurochem) were added, maintaining a molar ratio of acetic acid to methanol of 1:2. The reaction was conducted in a thermostatic bath with continuous stirring at 343 K for 4 h. The activity of the catalyst was estimated by titrating the post-reaction mixtures with 0.1 M sodium hydroxide solution to calculate the amount of unreacted acetic acid. Prior to analysis, the obtained mixtures were centrifuged to separate the catalyst from the reagent mixture. Subsequently, 0.5 cm^3^ of the resulting solution was transferred to a volumetric flask and diluted with 10 cm^3^ of water. Phenolphthalein was used as the indicator for the alkalimetric titration. The conversion of acetic acid *C_HAc_* (%) was calculated using the following equation:

$$C\%=\left(1-\frac{V}{V_{0}}\right)\cdot 100\%$$

where *V*_0_ and *V* are the volumes of NaOH solutions used for titration of the solution of substrates mixture before and after the reaction (mL), respectively.

The UV-Vis studies were conducted using the reflectance method on a Jasco V670 apparatus (ABL&E-JASCO Polska, Krakow, Poland). BaSO_4_ was used as an internal standard for recording UV-Vis DRS spectra. Measurements were performed at room temperature in the range 190–900 nm.

Transmission electron microscopy (TEM) images were recorded on a JEOL 2000 microscope (JEOL, Tokyo, Japan) operating at an accelerating voltage of 80 kV.

X-ray photoelectron spectroscopy (XPS) measurements were conducted using an Ultra High Vacuum (UHV) System (Specs, Berlin, Germany). The examined materials were irradiated with a monochromatic Al Kα radiation (1486.6 eV). The operating pressure in the chamber was close to 2 × 10^−9^ mbar. Binding energies (BE) were calibrated against the C1s peak originating from the carbon surface layer, set at 284.6 eV. Spectroscopic data were processed through CasaXPS software (version 2.3.22PR1.0) developed by Casa Software Ltd., Teignmouth, UK, employing a peak-fitting algorithm with a linear background correction.

The proton conductivity of the obtained composites was measured using impedance spectroscopy (EIS) in the temperature range of 300–393 K. Powder samples were pressed in a Teflon vessel between electrodes under a pressure of 200 MPa. The measurements were carried out in a nitrogen atmosphere using a Hewlett Packard 4284A precision LCR meter (20 Hz–1 MHz) (Keysight Technologies UK, Wokingham, UK). The measurement temperature was controlled and stabilized with a LakeShore 340 temperature controller (Lake Shore Cryotronics, Inc., Westerville, OH, USA).

## Figures and Tables

**Table 1:** The unit cell constant (a_0_), d-spacing values (d_211_), and the wall thickness (d_s_) for all matrices.

Sample	d_211_ [nm]	a_0_ [nm]	d_s_ [nm]
**H-KIT-6 (inf.)**	8.38	20.5	6.17
**H-KIT-6 (200)**	8.54	20.9	6.06
**H-KIT-6 (100)**	8.88	21.7	6.67
**H-KIT-6 (50)**	8.71	21.3	6.56
**H-KIT-6 (25)**	8.38	20.5	5.56
			
**Ce-KIT-6 (inf.)**	8.23	20.2	4.88
**Ce-KIT-6 (200)**	8.38	20.5	5.37
**Ce-KIT-6 (100)**	8.71	21.3	5.86
**Ce-KIT-6 (50)**	8.54	20.9	5.76
**Ce-KIT-6 (25)**	8.23	20.2	5.58

**Table 2:** The number of acid centers [μmol/g] estimated from the results of TPD-NH_3_.

Si/Al	25	50	100	200	Infinity
**H-**	95	57	27	30	21
**Ce-**	283	364	474	375	261

**Table 3:** Density of acid sites in the resulting matrices.

Si/Al	25	50	100	200	Infinity
H-KIT-6	0.10	0.06	0.03	0.04	0.03
Ce-KIT-6	0.40	0.56	0.70	0.60	0.40

Density of acidic centers [μmol/m^2^] calculated from the following formula: the total amount of acidic centers from TPD-NH_3_ measurements [μmol/g] divided by total surface calculated from BET method [m^2^/g].

**Table 4:** Catalytic activity in the esterification reaction of acetic acid with methanol.

	Sample	25	50	100	200	Infinity
Conv. of HAc [%]						
**H-KIT-6**		12.7	18.7	26.2	24.8	12.4
**Ce-KIT-6**		16.3	26.8	41.5	25.4	14.7

**Table 5:** Surface area (BET), pore volume, and average pore diameter of obtained KIT-6 materials.

Sample	S_BET_ [m^2^/g]	S_micro_ [m^2^/g]	S_ext_ [m^2^/g]	V_tot_ [cm^3^/g]	V_micro_ [cm^3^/g]	D [nm]
**H-KIT-6 (inf.)**	783	142	641	0.92	0.063	4.7
**H-KIT-6 (200)**	782	161	621	0.85	0.075	4.4
**H-KIT-6 (100)**	881	230	651	0.93	0.108	4.2
**H-KIT-6 (50)**	909	209	700	0.94	0.097	4.1
**H-KIT-6 (25)**	922	251	671	0.94	0.116	4.1
						
**Ce-KIT-6 (inf.)**	631	140	491	0.83	0.069	5.2
**Ce-KIT-6 (200)**	629	121	508	0.75	0.068	4.9
**Ce-KIT-6 (100)**	627	110	517	0.75	0.05	4.8
**Ce-KIT-6 (50)**	654	95	559	0.76	0.039	4.7
**Ce-KIT-6 (25)**	685	102	583	0.77	0.043	4.5

**Table 6:** Proton conductivity values of azole-loaded composites.

Compounds	Proton Carrier	Conductivity [S/cm]	E_a_ [eV]	Conditions	Reference
MOF-217	imidazole	1.1 × 10^−3^	0.58	anhydrous, 373 K	[74]
β-PCMOF2	1,2,4-triazole	2 × 10^−4^	0.51	anhydrous, 423 K	[75]
TPB-DMTP-COF	imidazole	4.37 × 10^−3^	0.38	anhydrous, 403 K	[76]
TPB-DMTP-COF	1,2,4-triazole	1.1 × 10 ^−3^	0.21	anhydrous, 403 K	[76]
CAU-11	imidazole	3.0 × 10^−4^	0.19	anhydrous, 383 K	[77]
BEA (zeolite)	imidazole	5.86 × 10^−4^	0.34	anhydrous, 393 K	[19]
FAU (zeolite)	imidazole	3.27 × 10^−4^	0.44	anhydrous, 393 K	[22]
[Al(µ_2_-OH)(1,4-ndc)]_n_	imidazole	2.2 × 10^−5^	0.6	anhydrous, 393 K	[7]
Td-PPI	imidazole	3.49 × 10^−4^	0.30	anhydrous, 363 K	[3]
PMO	imidazole	2.30 × 10^−4^	0.14	anhydrous, 453 K	[9]
s-PMO	imidazole	7.11 × 10^−3^	0.17	anhydrous, 453 K	[9]
PVA–SSA(S1)	imidazole	1.4 × 10^−3^	no data	anhydrous, 413 K	[11]
EB-COF	1,2,4-triazole	3.25 × 10^−3^	0.18	anhydrous, 433 K	[78]
PON-1	imidazole	5.2 × 10^−4^	0.4	anhydrous, 403 K	[79]
Ce-KIT-6 (100)	imidazole	1.13 × 10^−3^	0.31	anhydrous, 393 K	This work

**Table 7:** Weights of aluminum isopropoxide used for the synthesis of KIT-6 materials.

Sample	Al(izop.)_3_ [g]
H-KIT-6 (inf)	-
H-KIT-6 (200)	0.06
H-KIT-6 (100)	0.13
H-KIT-6 (50)	0.25
H-KIT-6 (25)	0.50

**Table 8:** Mass fractions and weights of imidazole used during the preparation of composites.

Mass Fraction	Imidazole [g]
0.29 Im	0.40
0.34 Im	0.52
0.4 Im	0.68
0.5 Im	1.00

## Data Availability

The data presented in this study are available on request from the corresponding author.

## Supplementary Materials

The following supporting information can be downloaded at: https://www.mdpi.com/article/10.3390/molecules29133239/s1, Scheme S1: The proposed concept of imidazole-Ce-KIT-6 composite formation; Figure S1: The TPD-NH_3_ profiles for indicated samples; Figure S2: PXRD patterns of indicated samples; Figure S3: Temperature dependence of the conductivity of Ce-KIT-6 (100) with different imidazole loading measured during the second cooling cycle; Figure S4: PXRD patterns of imidazole and selected composites with different imidazole loading.
