# Supplementary material for: Encapsulation of Imidazole into Ce-Modified Mesoporous KIT-6 for High Anhydrous Proton Conductivity
Source: Molecules. 2024 Jul 8;29(13):3239. doi: 10.3390/molecules29133239 (PMC11243413; doi:10.3390/molecules29133239)
Supplement: Supplementary file 1 [file molecules-29-03239-s001.zip › molecules-3091938-supplementary.pdf]

SUPPLEMENTARY MATERIAL

Encapsulation of imidazole into Ce-modified mesoporous KIT-6 for high anhydrous proton conductivity

Agata Tabero [1], Aldona Jankowska [1], Adam Ostrowski [2,*], Ewa Janiszewska [1], Jolanta Kowalska -Kuś [1], Agnieszka Held [1], Stanisław Kowalak [1,*]

[1] Faculty of Chemistry, Adam Mickiewicz University in Poznań, Uniwersytetu Poznańskiego 8, 61-614 Poznań, Poland
[2] Institute of Molecular Physics, Polish Academy of Sciences, Smoluchowskiego 17, Poznań, Poland

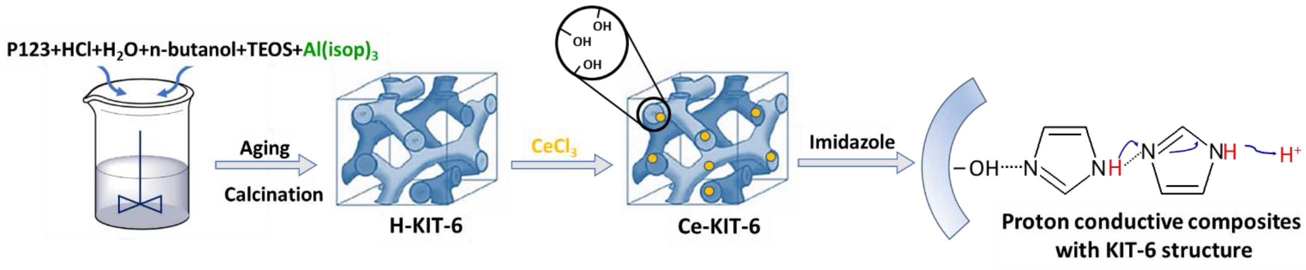

Scheme S1. The proposed concept of imidazole-Ce-KIT-6 composite formation.

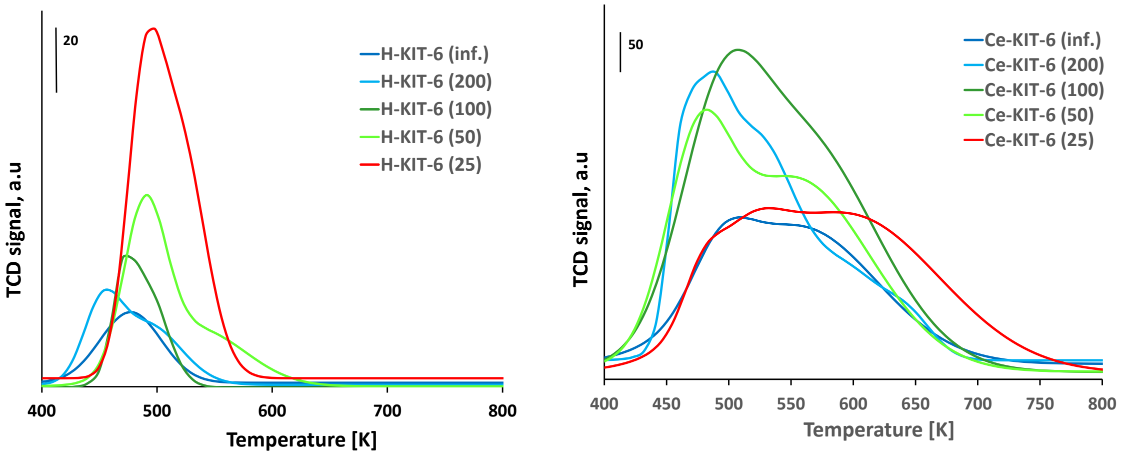

Figure S1. The TPD-NH_3 profiles for indicated samples.

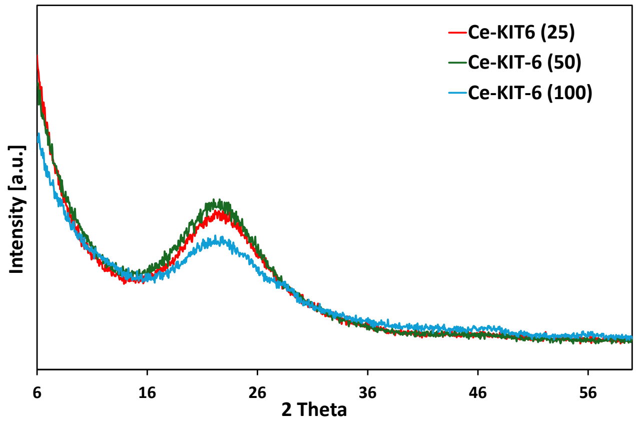

Figure S2. PXRD patterns of indicated samples.

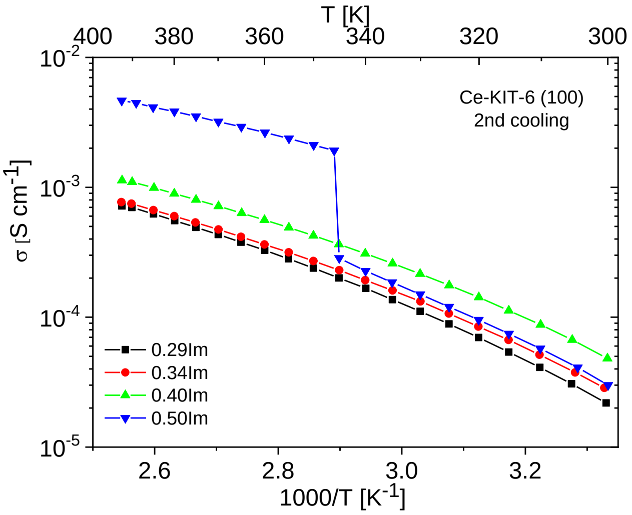

Figure S3. Temperature dependence of the conductivity of Ce-KIT-6 (100) with different imidazole loading measured during the second cooling cycle.

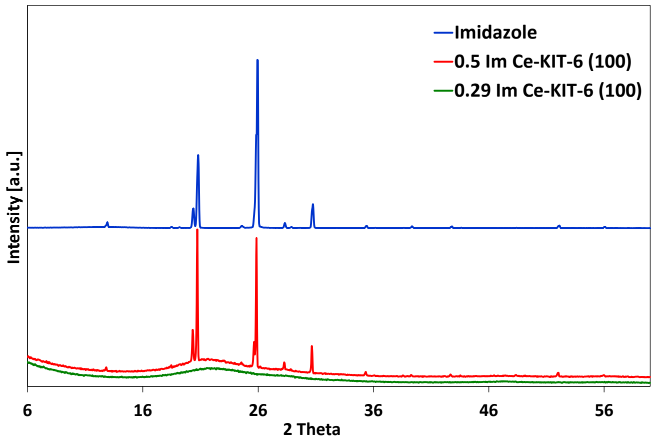

Figure S4. PXRD patterns of imidazole and selected composites with different imidazole loading.

***** Corresponding author; e-mail:

Stanisław Kowalak (skowalak@amu.edu.pl)
Adam Ostrowski (ostrowski@ifmpan.poznan.pl)
